# Fetal Thyroid Function, Birth Weight, and *in Utero* Exposure to Fine Particle Air Pollution: A Birth Cohort Study

**DOI:** 10.1289/EHP508

**Published:** 2016-09-13

**Authors:** Bram G. Janssen, Nelly D. Saenen, Harry A. Roels, Narjes Madhloum, Wilfried Gyselaers, Wouter Lefebvre, Joris Penders, Charlotte Vanpoucke, Karen Vrijens, Tim S. Nawrot

**Affiliations:** 1 Centre for Environmental Sciences, Hasselt University, Hasselt, Belgium; 2 Louvain Centre for Toxicology and Applied Pharmacology (LTAP), Université catholique de Louvain, Brussels, Belgium; 3 Biomedical Research Institute, Hasselt University, Hasselt, Belgium; 4 Department of Obstetrics, East-Limburg Hospital, Genk, Belgium; 5 Flemish Institute for Technological Research (VITO), Mol, Belgium; 6 Department of Clinical Biology, East-Limburg Hospital, Genk, Belgium; 7 Belgian Interregional Environment Agency, Brussels, Belgium; 8 Department of Public Health and Primary Care, Occupational and Environmental Medicine, Leuven University, Leuven, Belgium

## Abstract

**Background:**

Thyroid hormones are critical for fetal development and growth. Whether prenatal exposure to fine particle air pollution (≤ 2.5 μm; PM_2.5_) affects fetal thyroid function and what the impact is on birth weight in normal healthy pregnancies have not been studied yet.

**Objectives:**

We studied the impact of third-trimester PM_2.5_ exposure on fetal and maternal thyroid hormones and their mediating role on birth weight.

**Methods:**

We measured the levels of free thyroid hormones (FT_3_, FT_4_) and thyroid-stimulating hormone (TSH) in cord blood (*n* = 499) and maternal blood (*n* = 431) collected after delivery from mother–child pairs enrolled between February 2010 and June 2014 in the ENVIR*ON*AGE birth cohort with catchment area in the province of Limburg, Belgium.

**Results:**

An interquartile range (IQR) increment (8.2 μg/m^3^) in third-trimester PM_2.5_ exposure was inversely associated with cord blood TSH levels (–11.6%; 95% CI: –21.8, –0.1) and the FT_4_/FT_3_ ratio (–62.7%; 95% CI: –91.6, –33.8). A 10th–90th percentile decrease in cord blood FT_4_ levels was associated with a 56 g decrease in mean birth weight (95% CI: –90, –23). Assuming causality, we estimated that cord blood FT_4_ mediated 21% (–19 g; 95% CI: –37, –1) of the estimated effect of an IQR increment in third-trimester PM_2.5_ exposure on birth weight. Third-trimester PM_2.5_ exposure was inversely but not significantly associated with maternal blood FT_4_ levels collected 1 day after delivery (–4.0%, 95% CI: –8.0, 0.2 for an IQR increment in third-trimester PM_2.5_).

**Conclusions:**

In our study population of normal healthy pregnancies, third-trimester exposure to PM_2.5_ air pollution was associated with differences in fetal thyroid hormone levels that may contribute to reduced birth weight. Additional research is needed to confirm our findings in other populations and to evaluate potential consequences later in life.

**Citation:**

Janssen BG, Saenen ND, Roels HA, Madhloum N, Gyselaers W, Lefebvre W, Penders J, Vanpoucke C, Vrijens K, Nawrot TS. 2017. Fetal thyroid function, birth weight, and *in utero* exposure to fine particle air pollution: a birth cohort study. Environ Health Perspect 125:699–705; http://dx.doi.org/10.1289/EHP508

## Introduction

During prenatal life, thyroid hormones are critical for fetal growth and development, especially neurodevelopment (Burrow et al. 1994; Morreale de Escobar et al. 2004). Unbalanced thyroid function influences pregnancy outcomes and adversely affects the fetus. In particular, both maternal hypo- and hyperthyroidism are associated with increased risk of low birth weight (Blazer et al. 2003; Millar et al. 1994), whereas other studies also suggest an important role of fetal thyroid function in regulating fetal growth (Medici et al. 2013; Shields et al. 2011).

Thyroxine (T_4_), the major form of thyroid hormone, and triiodothyronine (T_3_), the active form, are controlled by thyroid-stimulating hormone (TSH) and released by the thyroid gland. Bound to plasma proteins, these thyroid hormones are transported throughout the body and diffuse from maternal blood across the placenta to reach the fetus (Calvo et al. 2002). However, it is the unbound, free fractions of these hormones (FT_4_ and FT_3_) that are taken up by different cell types to regulate their functioning (Hennemann et al. 2001). From the second trimester of gestation onward, the fetal thyroid gland becomes functional, and the fetus is able to produce its own supply of thyroid hormones in addition to the maternal supply (Morreale de Escobar et al. 2004).

Findings from previous studies suggest that airborne persistent organic pollutants (Abdelouahab et al. 2013; Baccarelli et al. 2008; Maervoet et al. 2007), cadmium (Iijima et al. 2007), and exposure to active and passive cigarette smoke (Soldin et al. 2009) may affect thyroid hormone regulation and function in neonates and adults; however, epidemiological studies on the impact of exposure to particulate matter (PM) air pollution on thyroid hormones are lacking. In large areas of the world, PM air pollution is an omnipresent environmental risk factor of public health concern, especially the fine particles with an aerodynamic diameter ≤ 2.5 μm (PM_2.5_). Exposure to ambient PM_2.5_ pollution during pregnancy has been found to be significantly associated with increased risk of low birth weight at term in mother–child cohorts of 12 European countries (Pedersen et al. 2013) and preterm birth (20–36 weeks of gestation) in a very large cohort of singleton pregnancies from three states of the United States (Rappazzo et al. 2014).

Despite the well-established link between PM_2.5_ air pollution and adverse gestational outcome (Pedersen et al. 2013), the role of fetal thyroid function in this association has never been investigated. Therefore, we hypothesized that airborne PM_2.5_ exposure during gestation affects fetal thyroid hormone function in normal healthy pregnancies and contributes to reduced birth weight. We tested this hypothesis in the framework of a mother–child cohort by studying the impact of third-trimester PM_2.5_ exposure on fetal and maternal thyroid hormone function, as reflected by the levels of FT_3_, FT_4_, and TSH in cord blood and maternal blood, and their mediating role on birth weight.

## Methods

### Study Population

From February 2010 through June 2014, we recruited 640 mother–child pairs after delivery at the East-Limburg Hospital in Genk, Belgium. They were enrolled in the on-going ENVIR*ON*AGE birth cohort study (ENVIRonmental influence *ON* early AGEing) following procedures previously approved by the Ethical Committee of Hasselt University and the East-Limburg Hospital (Janssen et al. 2012). The study was conducted according to the principles outlined in the Declaration of Helsinki for investigation of human subjects. The participation rate of eligible mothers in the birth cohort (mothers able to fill out a Dutch language questionnaire and those without planned cesarean section) was 61%, and enrollment was spread equally over all seasons of the year. Midwives recorded the reason for nonparticipation. The main reasons (in descending importance) were failure to ask for participation, communication problems, or complications during labor. Participating mothers provided written informed consent when they arrived at the hospital for delivery. They completed study questionnaires in the postdelivery ward to provide detailed information on maternal age, prepregnancy body mass index (BMI), maternal education, occupation, self-reported smoking status, alcohol consumption, place of residence, use of medication, parity, and newborn’s ethnicity. Former smokers were defined as those who had quit smoking before pregnancy. Smokers continued smoking during pregnancy. Based on the native country of the newborn’s grandparents, we classified his/her ethnicity as European-Caucasian when two or more grandparents were European, or non-European when at least three grandparents were of non-European origin. Maternal education was coded as “low” (no diploma or primary school), “middle” (high school), or “high” (college or university degree). After birth, we collected perinatal parameters from the medical files such as birth date, gestational age, newborn’s sex, birth weight and length, length of labor, Apgar score, pH of arterial cord blood, and ultrasonographic data.

**Figure 1:**
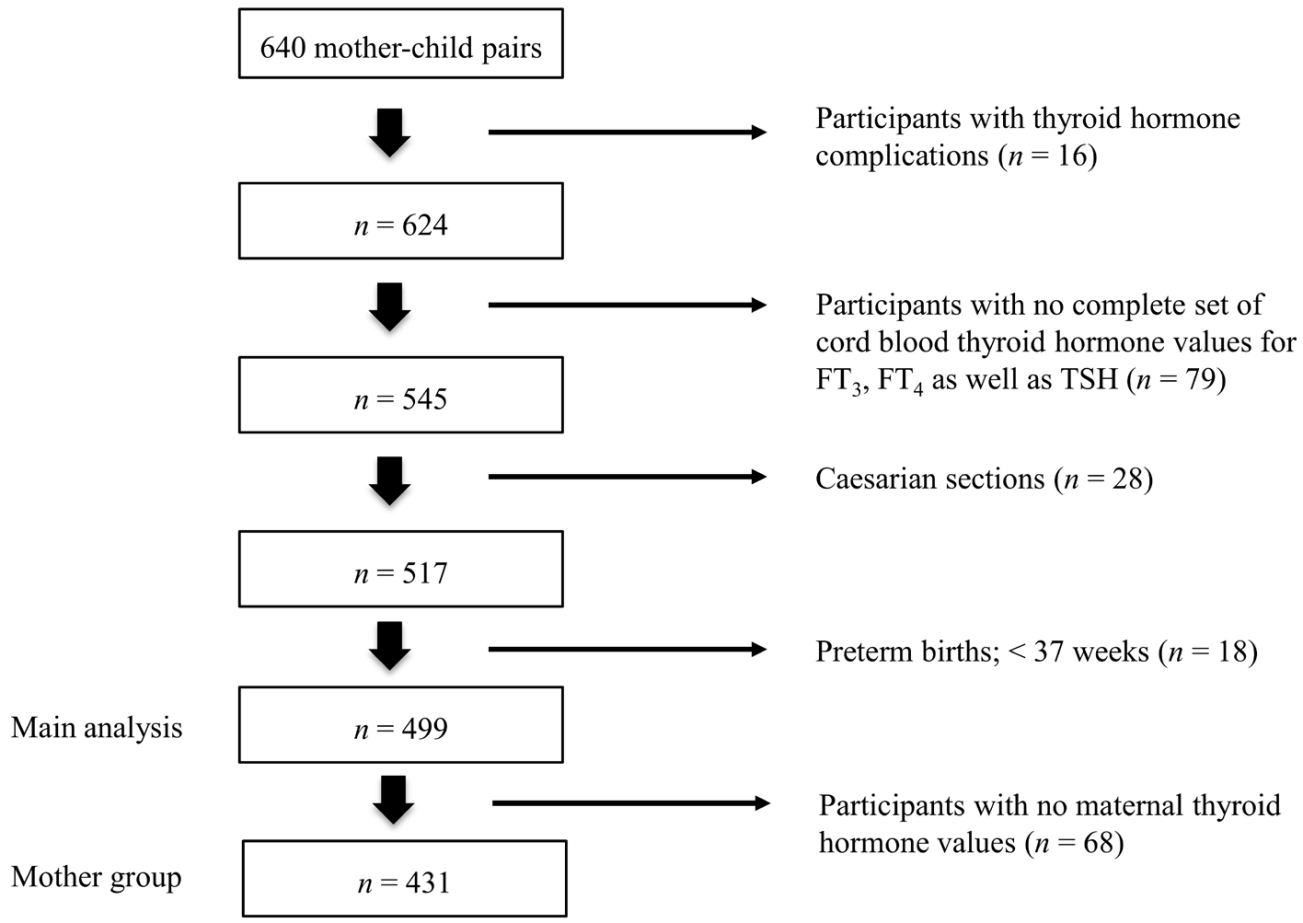
Flow chart depicting the selection procedure of study participants from the ENVIR*ON*AGE birth cohort, Limburg, Belgium.

The main analysis of our investigation was conducted in a subcohort of the 640 singleton pregnancies in the ENVIR*ON*AGE birth cohort, after excluding 16 mothers with hyper- or hypothyroidism, 79 mothers from whom we had no complete set of cord blood thyroid hormone values, 28 cesarean sections, and 18 preterm births (< 37 weeks), leaving 499 mother–child pairs for the main analysis (Figure 1). Additionally, maternal blood could not be collected from 68 mothers, resulting in a study population of 431 for the maternal thyroid hormone analysis (mother group) (Figure 1). Our study population was generally similar to all births in Flanders [data obtained from the Study Centre for Perinatal Epidemiology (SPE)] as to maternal age, education, parity, sex, ethnicity, and birth weight (see Table S1) (Cox et al. 2013).

### Ambient Exposure Assessment

For each mother’s residential address, we interpolated the regional background PM_2.5_ (μg/m^3^) using a spatial temporal interpolation method (kriging method) (Janssen et al. 2008) that uses pollution data collected by the official fixed-site monitoring network and land-cover data obtained from satellite images (CORINE land-cover data set) in combination with a dispersion model (Lefebvre et al. 2011). This model chain provides daily PM_2.5_ values using data from the Belgian telemetric air quality network and point and line sources, which are then interpolated in a high-resolution receptor grid. In the Flemish region of Belgium, the interpolation tool explained > 80% of the temporal and spatial variability (Maiheu et al. 2013). We defined the third trimester of pregnancy as from week 27 to delivery and calculated the mean PM_2.5_ values for this trimester. The date of conception was estimated on the basis of the first day of the mother’s last menstrual period, combined with the first ultrasound exam. Complete information for the residential address during pregnancy was obtained by questionnaire and checked with hospital records. For those who moved residence during pregnancy (*n* = 54; 10.8%), we calculated the third-trimester exposure window allowing for the changes in address during this period.

Mean daily temperatures and relative humidity for the study region were provided by the Royal Meteorological Institute (Brussels, Belgium). We calculated the third-trimester apparent temperature by using the following formula (Kalkstein and Valimont 1986; Steadman 1979): –2.653 + (0.994 × Ta) + (0.0153 × Td^2^), where Ta is air temperature and Td is dew point temperature (degrees Celsius).

### Blood Collection and Thyroid Hormone Measurements

Umbilical cord and maternal blood samples (8 mL each) were collected in plastic BD Vacutainer® Lithium Heparin Tubes (BD, Franklin Lakes, NJ, USA) immediately after delivery and 1 day after delivery, respectively. The samples were centrifuged (3,200 rpm for 15 min) to retrieve plasma which was instantly frozen at –80°C. The plasma levels of FT_4_ (pmol/L), FT_3_ (pmol/L), and TSH (mU/L) were measured with an electro-chemiluminescence immunoassay using the Modular E170 automatic analyzer (Roche, Basel, Switzerland) at the clinical laboratory of East-Limburg Hospital.

### Statistical Analysis

For database management and statistical analysis, we used the SAS software program (version 9.2; SAS Institute Inc., Cary, NC, USA). Thyroid hormone levels were log_10_-transformed to improve the normality of the distributions and described by geometric mean and 10th–90th percentile. The ratio FT_4_/FT_3_ was calculated using untransformed values and had a normal distribution. Pearson correlation coefficients were calculated among the different thyroid hormone levels in blood (FT_3_, FT_4_, and TSH) and between thyroid hormone levels and birth weight. We performed multiple linear regressions to assess the associations between newborn or maternal thyroid hormones and PM_2.5_ exposure during third trimester of gestation, and between newborn or maternal thyroid hormones and birth weight. Exposures to PM_2.5_ were fitted as linear variables in the models, and effect estimates on thyroid hormones were calculated for an interquartile range (IQR) increment in PM_2.5_. The effect estimates of cord blood FT_4_ on birth weight were calculated for a 10th–90th percentile decrease in FT_4_, which corresponds to an 11% difference in FT_4_. All cord blood models were adjusted for sex, gestational age (weeks), season of delivery [winter (21 December–20 March)/spring (21 March–20 June)/summer (21 June–20 September)/autumn (21 September–20 December)], Apgar score (< 9/9/10), maternal age (years), prepregnancy BMI (kg/m^2^), smoking status (never-smoker/cessation before pregnancy/smoker), parity (1/2/≥ 3), ethnicity (European-Caucasian, yes or no), maternal education (low/middle/high), and third-trimester apparent temperature (°C), and all models for maternal blood were adjusted for the same covariates except newborn’s sex and Apgar score. In an additional analysis, we adjusted the cord blood models for maternal thyroid hormones. The Shapiro–Wilk statistic and Q-Q plots of the residuals were used to test the assumptions of model linearity.

We used mediation analysis to investigate potential associations that may underlie the relation between the exposure variable (PM_2.5_) and the continuous outcome variable (birth weight, g) by examining how they relate to a third variable, the mediator (cord blood FT_4_ levels) (Valeri and Vanderweele 2013). We accomplished this by decomposing the total effect into a direct effect (DE; exposure effect on outcome at a fixed level of the mediator) and an indirect effect (IE; exposure effect on outcome that operates through the mediator). Mediation analysis is based on several assumptions: All associations are causal, with no uncontrolled confounding of associations between the exposure and mediator, the exposure and the outcome, or the mediator and the outcome; no measured mediator–outcome confounder is affected by exposure; and no interaction occurs between the exposure and mediator (Valeri and Vanderweele 2013).

### Sensitivity Analysis

Tobacco smoke exposure, a form of personalized airborne PM exposure, has been shown to influence maternal and fetal thyroid function through changes in thyroid hormone levels (Männistö et al. 2012; McDonald et al. 2008; Shields et al. 2009; Soldin et al. 2009). In a sensitivity analysis, we performed linear regression analysis to examine the associations between newborn or maternal thyroid hormones and smoking, adjusting for the same co-variables as mentioned above except smoking. Additionally, we repeated the analysis between cord blood thyroid hormones and PM_2.5_ exposure while excluding smokers.

Thyroid hormones may also show seasonal variations linked to changes in temperature (Reed 1995). To account for possible seasonal differences between subjects, we calculated for each subject an exposure window covering a 1-year period: 365 days calculated backward from the date of delivery.

Furthermore, it is known that cord blood thyroid hormone levels are influenced by different external factors. We explored whether covariates such as cord plasma estradiol (Lv et al. 2014), passive smoking (Soldin et al. 2009), alcohol consumption (Herbstman et al. 2008), pH of arterial cord blood (Chan et al. 2001), or length of labor (Parate et al. 2010), known for their interference with thyroid hormones, may alter the association between cord blood thyroid hormones and third-trimester PM_2.5_ exposure.

## Results

### Demographics of Participants

Table 1 shows demographic characteristics and perinatal traits of the mother–child group (*n* = 499). Mean (10th–90th percentile) maternal age was 29.1 years (23–35) and mean prepregnancy BMI was 23.9 (19.6–29.8) kg/m^2^. Most women never smoked (*n* = 316), 113 stopped smoking before pregnancy, and 70 mothers reported to continue with smoking during pregnancy (on average 8.6 cigarettes/day). More than 80% of the mothers reported no consumption of alcoholic beverages during pregnancy. The newborns, among them 254 girls (50.8%), had a mean gestational age of 39.4 weeks (38–41) and comprised 275 primiparous and 170 secundiparous newborns. About 90% of the newborns were Europeans of Caucasian ethnicity. The mean birth weight of the newborns was 3,446 (2,915–3,990) g. Five minutes after delivery, > 90% of the newborns had an Apgar score ≥ 9.

**Table 1:** Characteristics and thyroid hormone levels of the mother–child pairs (*n* = 499).

Characteristic	*n* (%) or mean (10th–90th percentile)
Mothers
Age (years)	29.1 (23–35)
Prepregnancy BMI (kg/m^2^)	23.9 (19.6–29.8)
Education^*a*^
Low	61 (12.3%)
Middle	182 (36.5%)
High	256 (51.2%)
Self-reported smoking status
Never-smoker	316 (63.2%)
Cessation before pregnancy	113 (22.7%)
Smoker during pregnancy	70 (14.1%)
Self-reported passive indoor smoking (*n* = 486)	43 (8.8%)
Alcohol consumption (*n* = 485)
None	398 (82.1%)
Occasionally	87 (17.9%)
Parity
1	275 (55.0%)
2	170 (34.1%)
≥ 3	54 (10.9%)
Newborns
Sex
Female	254 (50.8%)
European-Caucasian ethnicity^*b*^	435 (87.2%)
Gestational age (weeks)	39.4 (38–41)
Season of delivery
Winter (December–March)	142 (28.5%)
Spring (March–June)	113 (22.7%)
Summer (June–September)	107 (21.4%)
Autumn (September–December)	137 (27.4%)
Apgar score 5 min after birth
7 or 8	39 (7.8%)
9	140 (28.1%)
10	320 (64.1%)
pH of arterial cord blood (*n* = 431)	7.2 (7.2–7.3)
Birth weight (g)	3,446 (2,915–3,990)
Minutes of labor (*n* = 427)	27.3 (8–54)
Cord blood thyroid hormones
TSH, mU/L	10.3 (5.5–22.3)
FT_3_, pmol/L	2.5 (2.0–3.2)
FT_4_, pmol/L	15.7 (13.5–18.5)
Ratio FT_4_/FT_3_	6.4 (5.0–8.0)
Maternal thyroid hormones (*n* = 431)^*c*^	
TSH, mU/L	2.1 (1.1–4.0)
FT_3_, pmol/L	4.2 (3.4–5.1)
FT_4_, pmol/L	12.5 (10.0–15.2)
Ratio FT_4_/FT_3_	3.0 (2.4–3.8)
For TSH, FT_3_, and FT_4_ levels, the geometric mean (10th–90th percentile) is given. ^***a***^Mother’s education: low (no high school diploma), middle (high school diploma), high (college or university diploma). ^***b***^Based on the native country of the newborn’s grandparents. European-Caucasian when two or more grandparents were European, or non-European when at least three grandparents were of non-European origin. ^***c***^Total group minus mothers from whom blood samples were not available.	

### Thyroid Hormone Levels in Cord Blood and Maternal Blood

The geometric means of thyroid hormone levels in cord blood (*n* = 499) were 10.3 mU/L for TSH, 2.5 pmol/L for FT_3_, and 15.7 pmol/L for FT_4_, whereas in maternal blood (*n* = 431) it was 2.1 mU/L, 4.2 pmol/L, and 12.5 pmol/L respectively (Table 1). A positive correlation was observed between FT_3_ and FT_4_ (cord blood: *r* = 0.30; *p* < 0.0001; maternal blood: *r* = 0.27; *p* < 0.0001) and between FT_3_ and TSH (cord blood: *r* = 0.11; *p* = 0.01; maternal blood: *r* = 0.19; *p* < 0.0001). Maternal FT_4_ levels were positively correlated with cord blood FT_4_ levels (*r* = 0.21; *p* < 0.0001), whereas an inverse correlation was observed with cord blood FT_3_ levels (*r* = –0.11; *p* = 0.01). Compared with maternal values, the measured cord blood FT_3_ levels were approximately 2-fold lower and the TSH levels much higher. The thyroid hormone concentrations in cord blood were similar to values published by others (Abdelouahab et al. 2013).

### Ambient Exposure Levels

Average (25th–75th percentile) PM_2.5_ exposure and apparent temperature for the third gestational trimester were respectively 16.0 μg/m^3^ (11.6–19.8) and 8.7°C (3.2–14.7). Mean levels of both parameters were similar throughout the trimesters of pregnancy (data not shown).

### Thyroid Hormones and PM_2.5_ Exposure During Gestation

**Figure 2:**
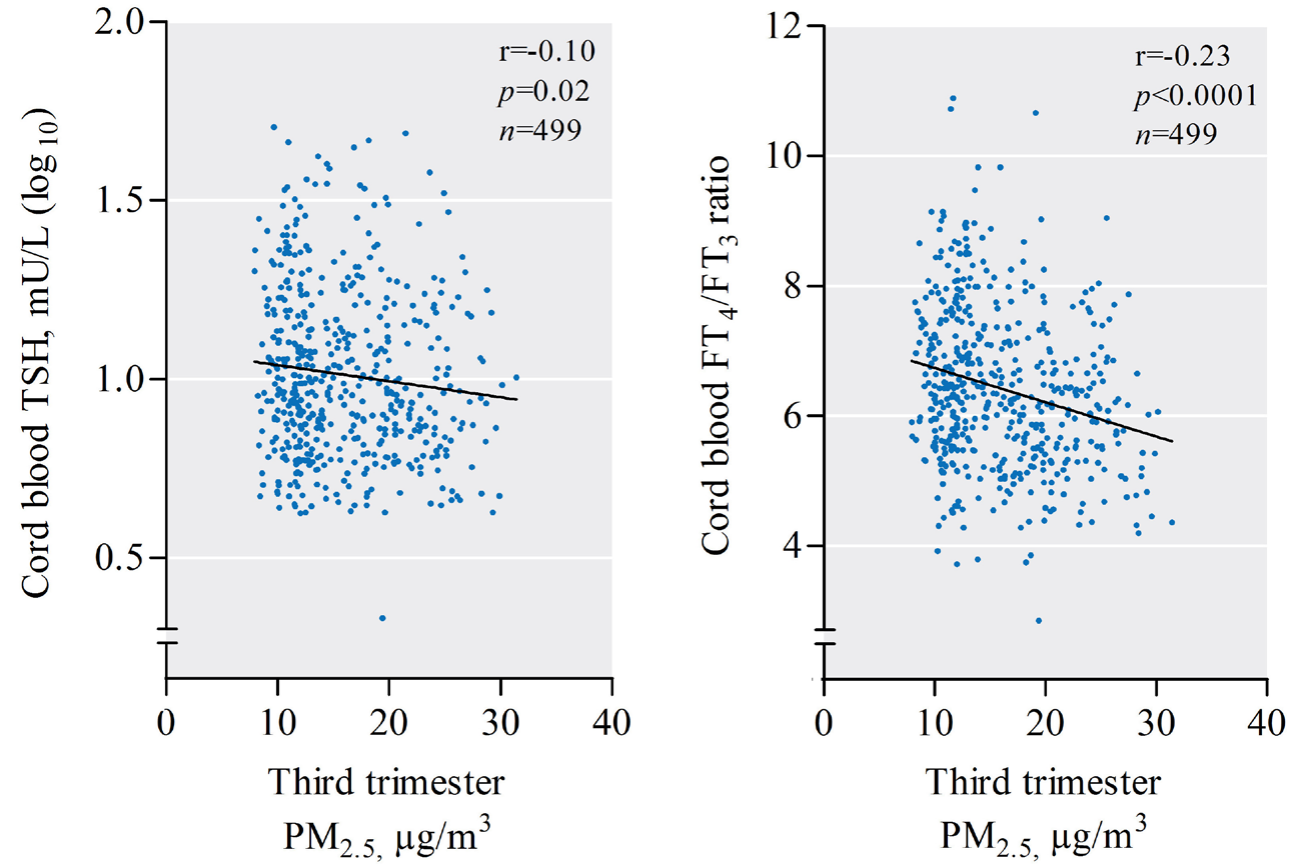
Unadjusted correlation between third-trimester PM_2.5_ exposure (μg/m^3^) and cord blood TSH (mU/L, log_10_) levels (left) and the FT_4_/FT_3_ ratio (right).

**Figure 3:**
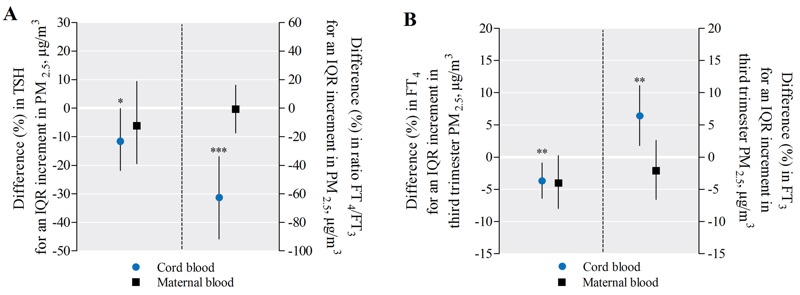
Difference in cord and maternal blood thyroid hormones in association with third-trimester PM_2.5_. The estimated relative difference in percentage (95% CI) is calculated for an IQR increment (8.2 μg/m^3^) in third-trimester PM_2.5_ exposure. Panel A displays the difference in TSH (left) and the difference in FT_4_/FT_3_ ratio (right). Panel B displays the difference in FT_4_ (left) and FT_3_ (right). The cord blood models were adjusted for sex, gestational age, season of delivery, Apgar score, maternal age, prepregnancy BMI, smoking status, parity, ethnicity, maternal education, and third-trimester apparent temperature, whereas for maternal blood sex and Apgar score were excluded. *p < 0.05. **p < 0.01. ***p < 0.001.

In cord blood (*n* = 499), TSH levels and FT_4_/FT_3_ ratios correlated inversely with PM_2.5_ exposure during the third trimester of pregnancy (Figure 2). After adjustment for sex, gestational age, season of delivery, Apgar score, maternal age, prepregnancy BMI, smoking status, parity, ethnicity, maternal education, and apparent temperature, an IQR increment (8.2 μg/m^3^) in PM_2.5_ exposure during the third trimester was associated with a lowering of 11.6% [95% confidence interval (CI): –21.8, –0.1; *p* < 0.05] in cord blood TSH levels (Figure 3A) and a lowering of 62.7% (95% CI: –91.6, –33.8; *p* < 0.0001) in cord blood FT_4_/FT_3_ ratio (Figure 3A). Considering the FT_4_ and FT_3_ levels in cord blood separately (Figure 3B), we observed opposite associations for an IQR increment of PM_2.5_ exposure on these two hormones during the third trimester (FT_4_, –3.7%; 95% CI: –6.4, –0.9; *p* = 0.009, and FT_3_, +6.4%; 95% CI: 1.8, 11.1; *p* = 0.006). Additional adjustment for maternal thyroid hormones in the cord blood models did not alter our findings for cord blood (data not shown).

In maternal blood (*n* = 431), TSH and FT_4_ levels correlated inversely with third-trimester PM_2.5_ exposure (*r* = –0.10; *p* = 0.04 and *r* = –0.13; *p* = 0.005 respectively). After adjustment for gestational age, season of delivery, maternal age, prepregnancy BMI, smoking status, parity, ethnicity, maternal education, and apparent temperature, only maternal FT_4_ levels were inversely but not significantly associated with an IQR increment in third-trimester PM_2.5_ exposure (–4.0%; 95% CI: –8.0, 0.2; *p* = 0.06) (Figure 3B). Neither TSH nor the FT_4_/FT_3_ ratio in maternal blood showed a significant difference with an IQR increment in third-trimester PM_2.5_ exposure.

### Thyroid Hormones and Birth Weight

After adjustment for gestational age and sex, neither FT_3_ nor TSH levels in maternal or cord blood were associated with birth weight (*p* ≥ 0.47). However, a 10th–90th percentile decrease (11%) in cord blood FT_4_ (log_10_ values) was associated with a lowering in birth weight of 71 g (95% CI: –103, –38; *p* < 0.0001). After additional adjustment for maternal age, prepregnancy BMI, smoking status, parity, season of delivery, Apgar score, ethnicity, maternal education, and apparent temperature, the association for the cord blood model remained significant (–56 g; 95% CI: –90, –23; *p* = 0.001). In contrast, a 10th–90th percentile decrease (15%) in maternal FT_4_ was positively associated with birth weight (45 g; 95% CI: 6, 83; *p* = 0.02), though the association was weaker and no longer significant after full adjustment (31 g; 95% CI: –7, 69; *p* = 0.11).

**Figure 4:**
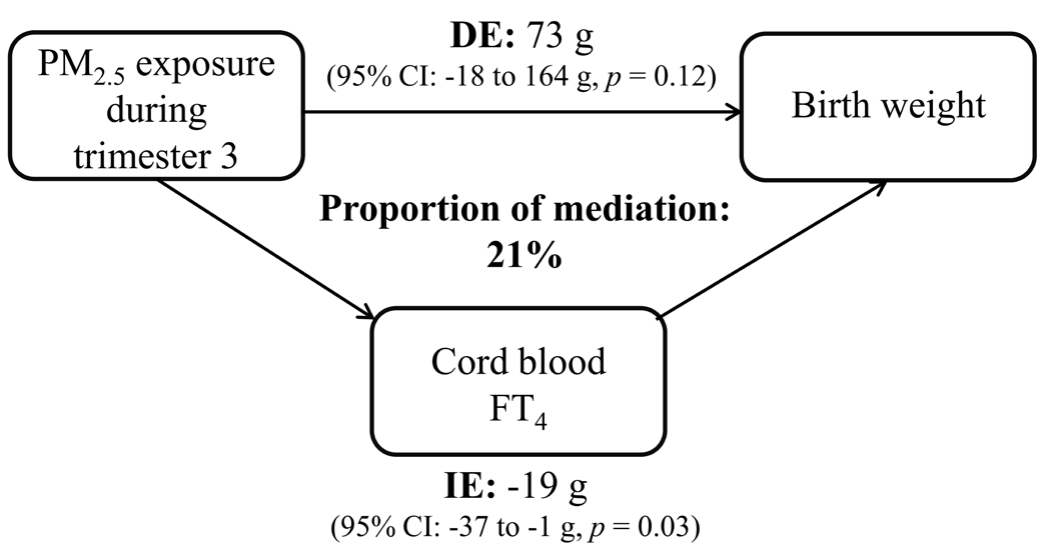
Estimated proportion of the association between PM_2.5_ exposure and birth weight (g) mediated by cord blood FT_4_ levels. The figure displays cord blood FT_4_ levels as mediator, the estimate of the indirect effect (IE), the estimate of the direct effect (DE), and the proportion of mediation (IE/DE + IE). The estimated effect is calculated for an IQR increment (8.2 μg/m^3^) in PM_2.5_ exposure during the third trimester of pregnancy. The mediation model was adjusted for sex, gestational age, season of delivery, Apgar score, maternal age, prepregnancy BMI, smoking status, parity, ethnicity, maternal education, and third-trimester apparent temperature.

We performed mediation analysis to estimate the proportion of the PM_2.5_ exposure effect on birth weight as mediated by cord blood FT_4_. Although we did not observe a significant association between third-trimester PM_2.5_ exposure and birth weight (*p* = 0.70), there is consensus among statisticians that the relationship between exposure (e.g., PM_2.5_) and outcome (e.g., birth weight) does not need to be statistically significant for a variable (e.g., FT_4_) to be a mediator (Valeri and Vanderweele 2013). Assuming causality, adjusted estimates of the proportion of mediation suggest that cord blood FT_4_ levels explained 21% (indirect effect: –19 g; 95% CI: –37, –1; *p* = 0.03) of the association between the third-trimester IQR PM_2.5_ exposure and birth weight (Figure 4). Because maternal thyroid hormones did not meet the assumptions for mediation (no association between maternal FT_4_ and birth weight), we did not perform a mediation analysis.

### Sensitivity Analysis

After adjustment for newborn’s sex, gestational age, season of delivery, Apgar score, maternal age, prepregnancy BMI, parity, ethnicity, and apparent temperature, cord blood TSH levels were lower in mothers who continued smoking during pregnancy (–18.7%; 95% CI: –29.1, –6.7; *p* = 0.003) and also in those who stopped smoking before pregnancy (–10.3%; 95% CI: –19.6, 0.1; *p* = 0.05) in comparison with never-smoking mothers (see Table S2). When excluding never-smokers, we observed, as expected, an inverse association between smoking years and TSH levels in cord blood (*r* = –0.21; *p* = 0.004; *n* = 173). We did not find differences in cord blood FT_4_ levels between smokers and former smokers compared with nonsmokers. Newborns from women who stopped smoking before pregnancy had slightly lower FT_3_ cord blood levels (–3.7%; 95% CI: –7.5, 0.1; *p* = 0.06), but, surprisingly, those who continued smoking had higher levels of cord blood FT_3_ (3.7%; 95% CI: –1.3, 9.0; *p* = 0.15) compared with nonsmokers. We did not find an association between maternal thyroid hormones and smoking status during pregnancy (*p* ≥ 0.31). The associations between cord blood thyroid hormones and PM_2.5_ exposures did not alter when women who smoked during pregnancy were excluded (data not shown).

The analysis to account for seasonal differences invariably showed for an IQR increment (3.7 μg/m^3^) of PM_2.5_ during a 1-year period inverse associations with cord blood TSH levels (–8.8%; 95% CI: –14.8, –2.4; *p* = 0.008), the FT_4_/FT_3_ ratio (–32.7%; 95% CI: –48.8, –16.6; *p* < 0.0001), and the FT_4_ levels (–1.7%; 95% CI: –3.2, –0.1; *p* = 0.03), and a positive association with FT_3_ levels (3.5%; 95% CI: 1.0, 6.0; *p* = 0.006), corroborating the associations found for the third trimester of pregnancy (Figure 3).

Additional adjustments for cord blood plasma estradiol (*n* = 498), passive indoor tobacco smoke exposure (*n* = 486), alcohol consumption (*n* = 485), or pH of arterial cord blood (indicator of hypoxemia) (*n* = 431) did not alter the associations between PM_2.5_ exposure and FT_3_ and FT_4_ thyroid hormones shown in the main analysis (see Table S3). Length of labor may influence thyroid hormone levels possibly due to the high energy demand during labor (Parate et al. 2010). Adjusting the main models for length of labor (*n* = 427) also did not alter the reported associations except that the association between cord blood TSH levels and third-trimester PM_2.5_ exposure was no longer significant (see Figure S1).

## Discussion

To the best of our knowledge, our study is the first to show associations between airborne PM_2.5_ exposure and cord blood thyroid hormones. A key finding is that PM_2.5_ exposure during the third trimester of gestation is inversely associated with TSH levels and the FT_4_/FT_3_ ratio in cord blood, but not with thyroid hormones in maternal blood. The FT_4_/FT_3_ ratio in cord blood is a useful indicator of how effectively the body is able to convert T_4_ into T_3_ (Yoshimura Noh et al. 2005). In addition, results of the mediation analysis suggested that cord blood FT_4_ is a partial mediator of the association between third-trimester pregnancy PM_2.5_ exposure and birth weight, assuming the underlying causal assumptions of mediation analysis are valid. Our findings highlight the potential influence of early-life environmental exposure to PM_2.5_ on fetal thyroid function and fetal growth. In addition, our results remained robust in multiple sensitivity analyses comprising maternal tobacco smoking, passive indoor smoking, seasonal variations, alcohol consumption, fetal hypoxemia, maternal estrogen levels, and length of labor.

During pregnancy, thyroid hormones regulate metabolism, stimulate differentiation and growth of the fetus, and influence neurocognitive development (Burrow et al. 1994; Morreale de Escobar et al. 2004). Despite the fact that the fetus starts secreting small amounts of thyroid hormone from mid-gestation onward (Thorpe-Beeston et al. 1991), the mother already supplies thyroid hormones to the fetal circulation from the first trimester without compromising her own supply (Vulsma et al. 1989). The rise of maternal thyroid hormones in the first trimester of pregnancy is considered critical to ensure normal (neurological) development (Morreale de Escobar et al. 2004). Maternal T_4_ and T_3_ diffuse across the placenta to reach concentrations in the fetus that are in the same range as those in adult tissues (Calvo et al. 2002). Fetal T_3_ is generated locally from T_4_ by type 2 deiodinase, has a high affinity for nuclear binding sites in the placenta, and stimulates the production of factors that control trophoblast growth and development (Maruo et al. 1991). This suggests that thyroid hormones play an important role in normal placentation and development of the fetus. Shields et al. (2011) showed in women with normal healthy pregnancies that placental weight was positively associated with cord blood FT_4_ levels and inferred that thyroid hormones may influence fetal growth indirectly by affecting placental growth. These authors found that lower FT_4_ levels in cord blood were associated with reduced birth weight, and their results are corroborated by our study and two studies from the Netherlands (Korevaar et al. 2016; Medici et al. 2013). Moreover, our study estimated that during the third trimester of pregnancy the estimated effect of PM_2.5_ exposure on birth weight was for 21% (on average –19 g) mediated by cord blood FT_4_ levels. As in all observational studies, these estimates should be interpreted with caution because the underlying assumptions of causality between each pair of factors in the mediation analysis cannot be verified. Nevertheless, this finding suggests that the third trimester of pregnancy, when the fetus significantly increases in size, is an important window of susceptibility to PM_2.5_ exposure. Shields et al. (2011), Medici et al. (2013), and León et al. (2015), as well as our study, report an inverse association between maternal FT_4_ and birth weight, which is opposite to cord blood FT_4_. In a study of pregnant women without history of thyroid dysfunction, it has been shown that lower concentrations of FT_4_ in maternal blood were related with increased placental growth (Bassols et al. 2011). These observations together suggest a functional discrepancy for FT_4_ between maternal and fetal blood, especially with regard to fetal growth. In our study, we observed an inverse but no significant association (*p* = 0.06) between maternal FT_4_ and third-trimester PM_2.5_ exposure in accordance with our findings in cord blood.

Contrary to maternal T_4_ and T_3_, perfusion experiments with TSH on human term placentas have shown that TSH crosses placental tissue and fetal membranes only sparingly (Bajoria and Fisk 1998). Hence, our finding of an inverse association between cord blood TSH levels and PM_2.5_ exposure during pregnancy suggests a potential effect of PM_2.5_ on fetal thyroid function. Experimental studies showed that PM exposure in healthy rats modulates the hypothalamic–pituitary–thyroid axis and leads to increases in markers of glucocorticoid activity (Thomson et al. 2013), which are known to suppress TSH release (Wilber and Utiger 1969). In the context of anti-inflammatory actions of glucocorticoids, previous findings from our birth cohort suggest that ambient PM_2.5_ exposure may induce a systemic oxidative stress response (Janssen et al. 2012) and increase placental protein-bound 3-nitrotyrosine (Saenen et al. 2016).

The FT_4_/FT_3_ ratio in cord blood, a useful indicator of how effectively the body is able to convert T_4_ into T_3_ (Bassols et al. 2011), was inversely associated with PM_2.5_ during pregnancy. This finding could be explained by the fact that placental type 2 deiodinase activity increases when the availability of T_4_ decreases, thus representing a potential homeostatic mechanism for maintaining T_3_ production when T_4_ concentrations are reduced (Glinoer 2004). In a population of 4,837 euthyroid Finnish mothers, Männistö et al. (2012) observed that mothers who smoked before, or continued smoking during the first trimester of pregnancy, had reduced blood levels of FT_4_ and increased levels of FT_3_ compared with nonsmokers. Constituents of tobacco smoke may stimulate the conversion of T_4_ to T_3_ in tissues by boosting type 2 deiodinase activity, as shown in cultured rat brain glial cells (Gondou et al. 1999). Low levels of TSH and FT_4_ are suggestive of central hypothyroidism (defect of thyroid hormone production due to insufficient stimulation by TSH of an otherwise normal thyroid gland) (Persani 2012). Recently, it has been shown that intrauterine exposure to insufficient maternal thyroid hormone levels, characterized by low levels of FT_4_ coexisting with reference TSH levels, was associated with higher scores for attention deficit/hyperactivity disorder (ADHD) symptoms in 127 children at 8 years of age of a population-based birth cohort in the Netherlands (Modesto et al. 2015). Additional research is needed to confirm our findings in other populations and to evaluate potential consequences later in life.

Our study has some limitations. First, thyroid hormones are responsive to environmental temperature (Reed 1995) and show a seasonal pattern, with lower values in the cold period than in the warm period of the year. Nevertheless, our results were robust for both seasonal differences between subjects as well as adjustment for third-trimester apparent temperature. Second, iodine is required for the synthesis of thyroid hormones, but we did not have information on iodine levels in maternal or cord blood. However, we excluded *a priori* clinically confirmed cases of hypo- and hyperthyroidism. Last, we acknowledge the fact that we cannot fully exclude residual or unmeasured confounding by other factors such as noise, polychlorinated biphenyls, heavy metals, or pesticides that could be associated with both ambient air pollution and thyroid function. Overall, the characteristics of the ENVIR*ON*AGE birth cohort were generally similar compared with all births in Flanders, except that we excluded cesarean sections and preterm births, so our findings might be generalizable to the gestational segment of the population at large (see Table S1). We used a standardized fine-scale exposure model for the estimation of residential fine particle air pollution levels of the pregnant mothers (on average 16.0 μg/m^3^) which are comparable with other European and U.S. cohorts with mean PM_2.5_ exposure values amounting to 18.5 (Pedersen et al. 2013) and 14.5 μg/m^3^ (Rappazzo et al. 2014), respectively.

## Conclusion

Our epidemiological finding of differences in fetal thyroid function in association with PM_2.5_ exposure is in line with the known effects of cigarette smoking on thyroid function during pregnancy (Männistö et al. 2012). Although confirmation in other study populations is needed, our findings suggest that cord blood FT_4_ may play a mediating role between PM_2.5_ exposure and birth weight during late pregnancy. The potential mechanisms and possible later-in-life adverse consequences are far from elucidated. Our findings are of critical public health importance because of the ubiquity of fine PM air pollution and the possibility of long-term health consequences of early-life alterations in thyroid function. Therefore, to promote a healthier living environment for children, our findings support a down-revision of the current European Union air pollution limit for PM_2.5_ of 25 μg/m^3^ (annual average threshold) in the direction of the World Health Organization–recommended limit of 10 μg/m^3^ (annual average) (World Health Organization 2006).

## Supplemental Material

(440 KB) PDF
