# Supplementary material for: Fetal Thyroid Function, Birth Weight, and in Utero Exposure to Fine Particle Air Pollution: A Birth Cohort Study
Source: Environ Health Perspect. 2016 Sep 13;125(4):699–705. doi: 10.1289/EHP508 (PMC5382000; doi:10.1289/EHP508)
Supplement: (440 KB) PDF [file EHP508.s001.acco.pdf]

Environ Health Perspect
DOI: 10.1289/EHP508

Note to readers with disabilities: *EHP* strives to ensure that all journal content is accessible to all readers. However, some figures and Supplemental Material published in *EHP* articles may not conform to 508 standards due to the complexity of the information being presented. If you need assistance accessing journal content, please contact ehponline@niehs.nih.gov. Our staff will work with you to assess and meet your accessibility needs within 3 working days.

Supplemental Material

Fetal Thyroid Function, Birth Weight, and *in Utero* Exposure to Fine Particle Air Pollution: A Birth Cohort Study

Bram G. Janssen, Nelly D. Saenen, Harry A. Roels, Narjes Madhloum, Wilfried Gyselaers, Wouter Lefebvre, Joris Penders, Charlotte Vanpoucke, Karen Vrijens, and Tim S. Nawrot

Table of Contents

Table S1. Characteristics of the ENVIR*ON*AGE birth cohort participants and all births from 2002-2011 in Flanders (Northern part of Belgium). 2

Table S2. Associations between smoking status during pregnancy and thyroid hormones in cord blood and maternal blood. 3

Table S3. Sensitivity analysis of the associations between an IQR increment (+8.2 µg/m^3) in third trimester $PM_{2.5}$ exposure and cord blood thyroid hormones. 4

Figure S1. Change in cord (●) and maternal (■) blood thyroid hormones in association with third gestational trimester $PM_{2.5}$, while adjusting for length of labor (n = 427). The estimated relative change in percentage (95% CI) is calculated for an IQR increment (+8.2 µg/m^3) in third trimester $PM_{2.5}$ exposure. Panel A displays the change in TSH (left) and the change in FT_4/FT_3 ratio (right). Panel B displays the change in FT_4 (left) and FT_3 (right). The cord blood models were adjusted for sex, gestational age, season of delivery, Apgar score, maternal age, pre-pregnancy BMI, smoking status, parity, ethnicity, maternal education, and apparent temperature, whereas for maternal blood sex and Apgar score were excluded. 5

Table S1. Characteristics of the ENVIR*ON*AGE birth cohort participants and all births from 2002-2011 in Flanders (Northern part of Belgium).

**Characteristic**	**ENVIR*ON*AGE** (***n*** **= 499)**	**Flanders**[a] (***n*** **= 606877)**
Maternal age, y	29.1 (23.0-35.0)	29.5 (23.5-35.8)
<25	16.2%	16.2%
25-35	76.8%	70.7%
>35	7.0%	13.1%
Pre-pregnancy BMI, kg/m^2	23.9 (19.6-29.8)	N/A
Maternal education		
Low	12.3%	13.1%
Middle	36.5%	40.8%
High	51.2%	46.1%
Parity		
1	55.0%	46.9%
2	34.1%	34.7%
≥3	10.9%	18.4%
Sex		
Male	49.2%	51.4%
Ethnicity		
European	87.2%	87.7%
Birth weight, g	3466 (2915-3990)	3360 (2740-3965)

Values are percentages or means (10th-90th percentiles).

[a] Cox B, Martens E, Nemery B, Vangronsveld J, Nawrot TS. 2013. Impact of a stepwise introduction of smoke-free legislation on the rate of preterm births: analysis of routinely collected birth data. BMJ 346:f441.

Table S2. Associations between smoking status during pregnancy and thyroid hormones in cord blood and maternal blood.

**Smoking status**[a]	**TSH**		**FT_3**		**FT_4**		**Ratio FT_4/FT_3**	
	**β**	**(95% CI)**	**β**	**(95% CI)**	**β**	**(95% CI)**	**β**	**(95% CI)**
**Cord blood (*n* = 499)**								
(Ref)	-	-	-	-	-	-	-	-
Self-reported cessation	-10.3	(-19.6, 0.1)[(*)]	-3.7	(-7.5, 0.1)	-0.5	(-3.1, 2.1)	20.0	(-6.4, 46.4)
Self-reported smoker	-18.7	(-29.1, -6.7)[**]	3.7	(-1.3, 9.0)	-1.0	(-4.1, 2.2)	-27.4	(-60.3, 56)
**Maternal blood (*n* = 431)**								
(Ref)	-	-	-	-	-	-	-	-
Self-reported cessation	9.2	(-4.1, 24.4)	3.2	(-0.9, 7.5)	-0.4	(-3.9, 3.3)	-8.4	(-22.6, 5.8)
Self-reported smoker	-1.9	(-16.7, 15.5)	2.7	(-2.5, 8.1)	0.1	(-4.4, 4.8)	-7.8	(-25.8, 10.2)

[a] β represents the percentage change in thyroid hormone level compared to the reference category (self-reported never-smokers). All models were adjusted for sex, gestational age, season of delivery, Apgar score, maternal age, pre-pregnancy BMI, parity, ethnicity, and apparent temperature, except in the models of maternal blood where sex and Apgar score were excluded. $^{(*)}p = 0.05$, $^{*}p < 0.05$, $^{**}p < 0.01$.

Table S3. Sensitivity analysis of the associations between an IQR increment (+8.2 μg/m^3) in third trimester $PM_{2.5}$ exposure and cord blood thyroid hormones.

Model variable	TSH		FT_3		FT_4		Ratio FT_4/FT_3	
	β	(95% CI)	β	(95% CI)	β	(95% CI)	β	(95% CI)
Main model	-11.6	(-0.1, -21.8)*	6.4	(1.8, 11.1)**	-3.7	(-0.9, -6.4)**	-62.7	(-91.6, 33.8)***
+ cord plasma estradiol (*n* = 498)	-11.6	(-0.4, -21.6)*	6.2	(1.7, 11.0)**	-3.8	(-1.0, -6.5)**	-62.3	(-91.2, -33.4)***
+ passive indoor smoke (*n* = 486)	-11.8	(-0.3, -22.0)*	6.5	(1.8, 11.4)**	-3.6	(-0.8, -6.4)*	-63.1	(-92.4, -33.9)***
+ alcohol consumption (*n* = 485)	-11.4	(0.3, -21.8)	6.1	(1.5, 11.0)**	-4.2	(-1.4, -7.0)**	-64.1	(-93.7, -34.6)***
+ pH of arterial blood (*n* = 431)	-8.4	(-19.3, 4.0)	6.8	(1.8, 12.1)**	-4.6	(-1.7, -7.4)**	-71.9	(-103.2, -40.6)***

The main model, adjusted for sex, gestational age, season of delivery, Apgar score, maternal age, pre-pregnancy BMI, smoking status, parity, ethnicity, maternal education, and third trimester apparent temperature, was additionally adjusted for each listed covariate in a separate model. $^{*}p < 0.05$, $^{**}p < 0.01$, $^{***}p < 0.001$.

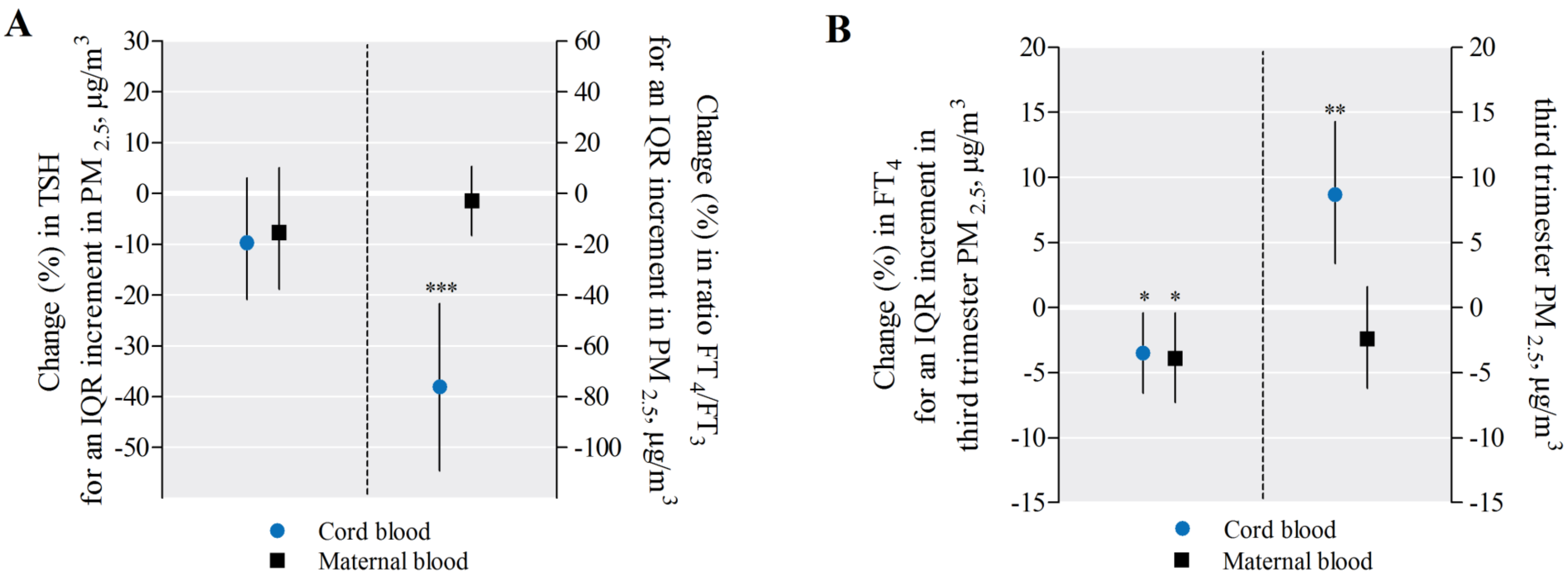

Figure S1. Change in cord (●) and maternal (■) blood thyroid hormones in association with third gestational trimester $PM_{2.5}$, while adjusting for length of labor (n = 427). The estimated relative change in percentage (95% CI) is calculated for an IQR increment (+8.2 µg/m^3) in third trimester $PM_{2.5}$ exposure. Panel A displays the change in TSH (left) and the change in FT_4/FT_3 ratio (right). Panel B displays the change in FT_4 (left) and FT_3 (right). The cord blood models were adjusted for sex, gestational age, season of delivery, Apgar score, maternal age, pre-pregnancy BMI, smoking status, parity, ethnicity, maternal education, and apparent temperature, whereas for maternal blood sex and Apgar score were excluded. $^{*}p < 0.05$, $^{**}p < 0.01$, $^{***}p < 0.001$.
